# Nonsteroidal Anti-Inflammatory Drugs Decrease Coagulopathy Incidence in Severe Burn Patients

**DOI:** 10.3390/ebj5020009

**Published:** 2024-04-28

**Authors:** Lyndon Huang, Kassandra Corona, Kendall Wermine, Elvia Villarreal, Giovanna De La Tejera, Phillip Howard Keys, Alen Palackic, Amina El Ayadi, George Golovko, Steven E. Wolf, Juquan Song

**Affiliations:** 1 School of Medicine, University of Texas Medical Branch, Galveston, TX 77555, USA; lghuang@utmb.edu (L.H.); kkcorona@utmb.edu (K.C.); kewermin@utmb.edu (K.W.); ellvilla@utmb.edu (E.V.); gidelate@utmb.edu (G.D.L.T.); phkeys@utmb.edu (P.H.K.); 2 Department of Hand, Plastic and Reconstructive Surgery, Burn Center, BG Trauma Center Ludwigshafen, University of Heidelberg, Ludwig-Guttmann-Str. 13, 67071 Ludwigshafen, Germany; alpalack@utmb.edu; 3 Department of Surgery, University of Texas Medical Branch, Galveston, TX 77555, USA; amelayad@utmb.edu (A.E.A.); swolf@utmb.edu (S.E.W.); 4 Department of Pharmacology, University of Texas Medical Branch, Galveston, TX 77555, USA; gegolovk@utmb.edu

**Keywords:** retrospective study, TriNetX database, international normalized ratio (INR), sepsis, mortality

## Abstract

The study investigated the impact of nonsteroidal anti-inflammatory drugs (NSAIDs) on burn-induced coagulopathy in severely burned patients. Patients with a greater than 20% TBSA were identified in the TriNetX research network and categorized into receiving or not receiving NSAIDs in the first week after the burn. The statistical significance of the rate of burn-induced coagulopathy, mortality and sepsis in the week following injury was analysed. We observed 837 severely burned patients taking NSAIDS during the week following the burn and 1036 patients without. After matching for age, gender and race, the risk of burn-induced coagulopathy significantly decreased (*p* < 0.0001) in patients taking NSAIDs (17.7%) compared to those without (32.3%). Patients taking NSAIDs were also less likely to develop sepsis (*p* < 0.01) and thrombocytopenia (*p* < 0.001) or die the week following injury (*p* < 0.0001). In conclusion, the early protective effects of NSAIDs at reducing the risk of coagulopathy as well as sepsis and mortality occur during the acute phase of burns.

## 1. Introduction

Burn injuries are common, with an occurrence rate of 5/100,000 people per year globally [1]. Major burns involving a greater than 20% total body surface area (TBSA) lead to elevated immune and inflammatory responses as well as systemic metabolic changes, which persists for years [2,3]. Without proper treatment, patients with massive skin barrier loss develop hypovolemic and distributive shock that can lead to multiple-organ failure and, ultimately, death [4]. Though the trends of burn incidence and mortality descend with the current care management improvements, patient life quality and complications related to health spending are even more concerning [5]. Every year, over 50,000 people experience burn injuries with hospitalisation, with over 20,000 having severe burns [6]. The cost of burn injury treatment is over USD 1 billion annually in the US, which does not include indirect costs of rehabilitation following the initial treatment [7]. The burn injury itself can lead to many other complications, including, but not limited to, disseminated intravascular coagulation (DIC), sepsis, nerve damage, acute respiratory distress syndrome, scarring and renal failure.

Recent advances in our understanding of coagulopathy have emphasized the dynamic balance between procoagulant pathways responsible for clot formation and the mechanisms that inhibit clot propagation beyond the site of injury [8]. This equilibrium is crucial for maintaining haemostasis and preventing either thrombosis or bleeding, which can be disrupted in critical illnesses or perioperative periods due to various factors. Haemostasis is often impaired following a severe burn injury, leading to the development of coagulopathy [9] and uncontrolled bleeding, which can lead to death. The impaired synthesis or depletion of coagulation factors may contribute to haematologic failure, which is a complex process. As such, patients who develop coagulopathy can be effectively treated with infusions of fresh frozen plasma and cryoprecipitate to replenish coagulation factors [10]. The biochemical processes involved in physiological haemostasis undergo regulation at multiple levels. This regulation is present at various stages of the coagulation cascade and some control mechanisms are activated based on various factors [11]. These control mechanisms are essential, as they prevent the coagulation cascade from becoming overactive, which can lead to inappropriate clot formation at the wrong place and time. Coagulopathy generally occurs when procoagulants are depleted and when the control mechanisms become disrupted. During coagulopathy, thrombin levels may be particularly low and thrombin may be improperly localized.

Recent studies highlighted the importance of the early diagnosis and management of burn-induced coagulopathy. For instance, Geng et al. (2020) explored the incidence and prognostic significance of acute burn-induced coagulopathy in patients with extensive burns, underscoring the strong predictive value of early-phase acute burn-induced coagulopathy for mortality [12]. Younan et al. (2017) identified that early coagulopathy in burn patients is significantly associated with an increased incidence of ventilator-associated events such as ventilator-associated pneumonia, indicating that the early recognition and management of coagulopathy could potentially reduce the occurrence of ventilator-associated events, thus, improving patient outcomes [13]. This research emphasizes the benefit of a prompt and accurate assessment of coagulation status in burn patients to improve outcomes.

A recent study revealed that the higher risk of coagulopathy with a larger size of burn (%TBSA) deserves comprehensive understanding for the clinical management of severely injured patients [14]. As far as we know, the development of coagulopathy is a result of clot formation abnormalities from the inflammatory and fibrinolytic mechanisms initiated after the burn [15]. This is most prevalent during the first 48 h, in which many pro-inflammatory molecules are upregulated in a hypermetabolic state [16]. This hypermetabolic phase is characterised by decreased vascular permeability, along with an increased heart rate and decreased vascular resistance, leading to a rise in cardiac output. In addition, infection, particularly sepsis, along with excision and grafting, can further affect coagulation by causing platelet dysfunction [17]. Burn-induced coagulopathy complicates an already precarious situation for many patients. Patients who develop coagulopathy are at an increased risk for mortality and morbidity [18]. Unfortunately, to date, information is scarce regarding the treatment or prevention of burn-induced coagulopathy.

The initial phase of burn resuscitation is characterized by intravascular volume depletion, oedema and increased vascular permeability [19]. Elevations in microvascular permeability result from both direct vascular thermal damage and the release of inflammatory mediators. This phase lasts for anywhere from 1 to 3 days. Intravascular hypovolemia and subsequent haemoconcentration ensue from the significant oedema formation within the initial 12 to 24 h after injury. Resuscitation is especially key during this phase to preserve tissue perfusion and to prevent ischemia from hypovolemia and cellular shock. The Parkland formula is commonly used to determine fluid resuscitation volume to assure haemodynamic stability. However, excessive volumes of fluid resuscitation can also contribute to the development of coagulopathy in these patients [14].

Nonsteroidal anti-inflammatory drugs (NSAIDs) are commonly prescribed in burn patients to relieve pain and reduce inflammation [20]. These are known to have effects on coagulation through their impact on platelets by inhibiting cyclooxygenase enzymes to decrease levels of thromboxane A2 [21]. Cyclooxygenase is required to convert arachidonic acid into thromboxanes, prostaglandins and prostacyclins. Furthermore, NSAIDs have anti-inflammatory effects, which could affect the activity and levels of pro-inflammatory molecules that lead to clot dysfunction and, ultimately, coagulopathy [22]. NSAIDs are often available as oral tablets and are usually well-absorbed from the gastrointestinal tract, with bioavailability often affected by the drug’s formulation. Topical NSAIDs are also an option and are useful for treating pain from soft tissue injuries [23]. These bypass the gastrointestinal system, offering localised relief with reduced systemic absorption and potentially fewer systemic side effects [24]. Finally, NSAIDS can be administered parentally; intravenous ibuprofen is often used and given as an infusion as a non-opioid analgesic to reduce inflammation and manage pain. Once absorbed, NSAIDs are widely distributed throughout the body. These are generally highly protein-bound, primarily to albumin, which affects their distribution volume [25]. The degree of protein binding can influence an NSAID’s efficacy and drug interactions. For example, high protein binding can lead to drug displacement interactions, potentially increasing the risk of toxicity. NSAIDs are primarily metabolized in the liver through cytochrome P450 enzymes and other pathways. The metabolic process can yield active or inactive metabolites, influencing the drug’s overall effect and duration of action. For instance, the metabolism of celecoxib involves CYP2C9, with genetic variations in this enzyme affecting the drug’s plasma levels and risk of adverse effects [26]. Genetic factors can also influence the metabolism of NSAIDs, leading to variability in drug response and risk of adverse effects among certain individuals.

The vascular response to NSAIDs was reported in 1984, which involves a complex interplay of effects on prostaglandin synthesis, blood pressure regulation, platelet function, endothelial health and the balance of vasoactive substances [27]. However, little is known of them impacting haemostatic coagulation following a severe burn. These anti-inflammatory mechanisms result in NSAIDs being a particularly appealing candidate drug to examine. Since clinical treatments are often medicated with a combination of treatments, we hypothesize that NSAIDs alleviate burn-induced coagulopathy by soothing the hyperinflammatory response. This retrospective study aims to analyse the impact of NSAIDs in the development of coagulopathy in severe burn patients by using a large database with real-world evidence. In addition, we also investigate other relevant outcomes for patients who receive NSAID treatment following injury, including the risk of DIC, sepsis, thrombocytopenia, purpura and death.

## 2. Materials and Methods

### 2.1. Data Resource

The patient data for this study were drawn from the TriNetX database in May 2021. The database (https://live.trinetx.com/) is a North American federated health research network providing global access to electronic medical records from over 69 million patients in 51 health-care organizations (HCOs). TriNetX is certified and maintains an information security management system (ISMS) to ensure the protection of the health-care data to which it has access and to meet the requirements of the HIPAA security rule. Data displayed on the TriNetX Platform follow the de-identification standard in Section §164.514(a) of the HIPAA privacy rule. The de-identified data process was attested to through a formal determination by a qualified expert in TriNetX, LLC, as defined in Section §164.514(b)(1) of the HIPAA privacy rule. The platform provides access to statistical summaries and aggregates counts in demographics, diagnoses, procedures, labs, outcomes and genomics. The study did not require any protected health information and personal data, so IRB approval was not needed (UTMB IRB exemption #20-0085).

### 2.2. Patient Populations

**Figure 1:**
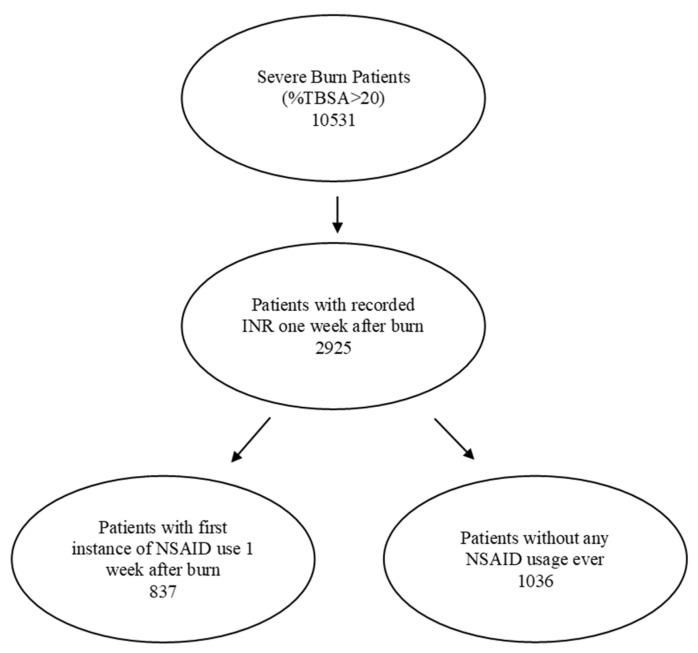
Study flows of patient population selection.

Severely burned patients were identified from 40 health-care organizations (HCOs) in the United States using ICD codes T31.2-T31.9, which encompassed 10,531 patients with burns involving more than 20% of their total body surface area. Of these, 2925 patients had an internationally normalised ratio of protime (INR) in the week following the burn injury. The patients were further categorized by whether or not they had received NSAIDs during the first week following the burn injury. The flow chart of the selected patient population is shown in Figure 1.

Among those receiving NSAIDs, ibuprofen (CN104-5640) and aspirin (BL117-1191) were the most prescribed at rates of 79% and 39%, respectively. The percentage of patients on each NSAID indicated that some patients were taking multiple NSAIDs (Table 1). Only patients whose first recorded instance of taking NSAIDs during that week were included. The following NSAIDs were included in this study: ibuprofen, oxaprozin, indomethacin, aspirin, diclofenac, celecoxib and naproxen.

### 2.3. Patient Outcomes with Statistical Analysis

Descriptive statistics were applied to NSAID usage in severe burn patients. The demographic information was further analysed using chi-squared tests. An elevated INR (>1.5) on hospital admission was a risk factor for mortality and morbidity after acute severe trauma, while an INR > 1.2 was not associated with an increased risk for the studied outcomes [28]. The interquartile range (IQR) for our study population was 1.2 (1.0–1.4) and the INR of 1.5 fell outside the third quartile. Thus, we defined the patient population with a threshold of international normalized ratio (INR) levels ≥ 1.5 as burn-induced coagulopathy throughout the week, with the most recent INR value being used. We then investigated NSAID use and comorbidity risk of coagulopathy in relation to %TBSA in severe burns using a linear regression analysis. Moreover, the statistical significance of the rate of burn-induced coagulopathy in the week following injury between the two groups was analysed using measures of association along with other outcomes, including sepsis (ICD code A41.9), thrombocytopaenia (ICD code D69.6) and mortality (ICD code R99). Provided by the TriNetX analytic tool package, we performed outcome measures of association, including the comparison of risk ratio with 95% CI, risk difference with 95% CI and t-test, odds ratio with 95% CI. Statistical significance was accepted at *p* < 0.05. The patients were 1:1 propensity-matched for age, gender and race for the outcome analysis.

## 3. Results

### 3.1. Demographics of Severe Burn Patients with NSAID Usage

From the TriNetX database, we identified 837 severely burned patients taking NSAIDs during the first week after injury without prior use and 1036 severely burned patients without any NSAID use with a recorded lab value of INR in the week following admission. The relevant demographic information is listed in Table 2.

When comparing the demographics of the two groups of severe burn patients, age was found to be a significant factor, with the average age of patients taking NSAIDs at 41 ± 21.6 and 46.6 ± 21.9 years old for those who were not (*p* < 0.0001). White race (60%) and males (72%) dominated in severe burn patients. We found no significant difference in the gender and race distribution of patients between the two groups. We found 2% more of Hispanic severely burned patients with NSAID treatment (*p* < 0.05).

**Figure 2:**
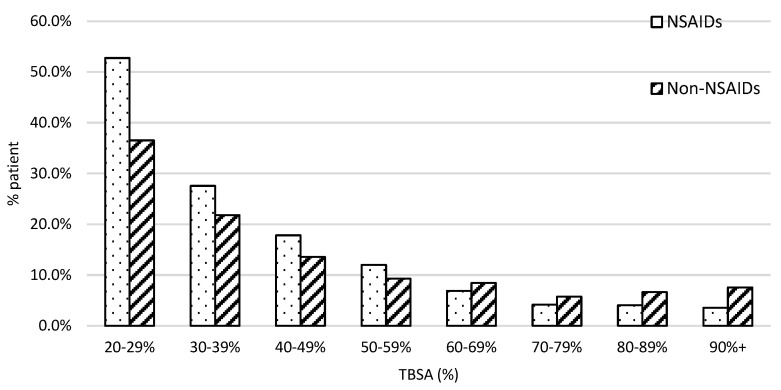
The patient distribution along with the injury severity. X-axis was stratified with every 10% interval from 20 to 100% TBSA. The dot bar stands for patients with NSAID treatment and the slant bar for those without.

The patient population with burn severity followed the typical first-order distribution in both groups, as presented in Figure 2. We found a greater proportion of patients who fell in the lower ranges of severe burn TBSA% than in the higher ranges. For example, 52% of patients received NSAIDs and 38% of patients did not who were in the 20–29% %TBSA range. However, the percentage of patients who were in the 90 + % TBSA range was under 10%.

#### Severe Burn Patients with NSAID Usage with Comorbidity Risk of Developing BIC

**Figure 3:**
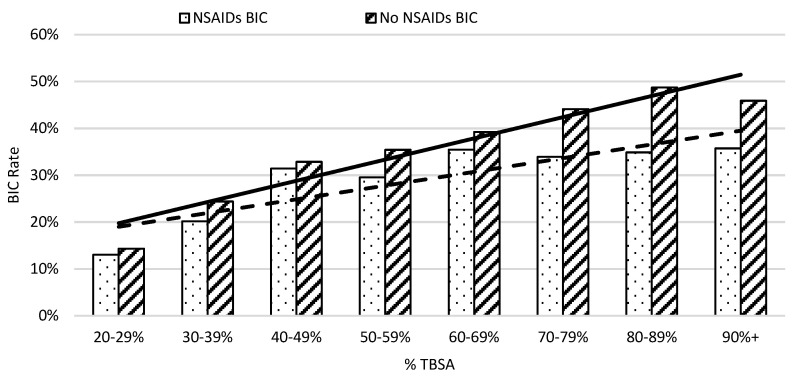
The risk of coagulopathy development following severe burns increased with %TBSA in 10% intervals. The x-axis represents burn size of the %TBSA and the y-axis shows the rate of burn patients with coagulopathy defined as an INR value greater than 1.5.

On average, in week one, patients not taking NSAIDs had an elevated risk of developing coagulopathy compared to those who did in 10% TBSA intervals (Figure 3). The risk of burn-induced coagulopathy increased from 13% to 30% in patients with NSAID treatment when burn severity increased from 20% to 59%; then, the risk of burn-induced coagulopathy rate remained at approximately 35% thereafter. In contrast, the risk of burn-induced coagulopathy was over 40% in severe burn patients, with greater than 69% TBSA burns when not receiving NSAIDs. The linear regression analysis showed that the greater slope of the trendline of patients not on NSAIDs also indicated a worse prognosis as %TBSA increased when compared to patients given NSAIDs. A linear regression analysis of burn-induced coagulopathy risk for an increasing %TBSA demonstrated a straight trend line with the equation of y = 0.0453x + 0.1525 (r2 = 0.9061) for patients not taking NSAIDs and a dot trend line with the equation of y = 0.0293x + 0.1607 (r2 = 0.7428) for patients taking NSAIDs. The steeper increased rate of burn-induced coagulopathy as the %TBSA increased in patients not taking NSAIDs indicated a worse prognosis.

**Figure 4:**
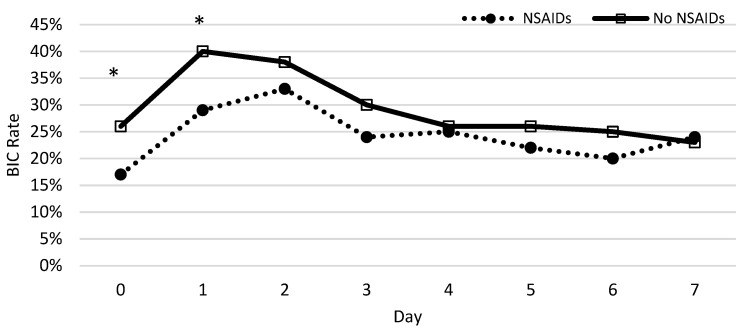
The rate of coagulopathy development following severe burns throughout the week. The x-axis represents the time after injury (day) and the y-axis shows the rate of burn patients with coagulopathy defined as an INR value greater than 1.5. The asterisks indicate the days on which NSAIDs decreased the significant risk of BIC using a chi-squared test.

The percentage of patients who developed coagulopathy in both groups was measured each day during the first week and a chi-squared test was used to test for significance. Significant differences between patients with and without NSAID prescriptions were observed on the same day and first day following the burn (Figure 4). The protective nature of NSAIDs was significantly demonstrated, with the greatest effect on the same day (*p* = 0.0002) and the first day following the burn (*p* = 0.0026). We found no significant difference from days 2 to 7.

After matching for age, gender and race, the risk of burn-induced coagulopathy significantly decreased (*p* < 0.0001) in patients taking NSAIDs (17.7%) compared to patients not taking NSAIDs (32.3%). We previously speculated that NSAIDs decreased burn-induced coagulopathy risk by reducing the systemic inflammation response. As for clinical pain management, acetaminophen is often prescribed, which is not an anti-inflammatory and non-opioid analgesic drug. In addition, acetaminophen has similar pharmacological properties to NSAIDs, both inhibiting COX-1/2 enzymes [29], but little inflammatory action [30]. When compared to patients taking the analgesic acetaminophen as a negative control, we found that the risk of developing coagulopathy still remained lower in patients taking the NSAIDs listed in this study for the first week (*p* < 0.0001) (Table 3). The results further implied the specific role of NSAIDs decreasing burn-induced coagulopathy risk by alleviating the inflammatory response, rather than through pain control management or other mechanisms.

Furthermore, patients taking NSAIDs were less likely to develop sepsis (*p* = 0.0046) and thrombocytopaenia (*p* = 0.0003) and die the week following injury (*p* < 0.0001). Other findings that supported the protective nature of NSAIDs against coagulopathy were a decreased risk of developing disseminated intravascular coagulation (DIC) (*p* = 0.0153) and purpura (*p* < 0.0001) one week following burn injury. We found no increased risk of stroke or haematuria in patients taking NSAIDs one week following injury (Table 4).

## 4. Discussion

The protection that NSAIDs offer in the development of burn-induced coagulopathy was demonstrated throughout various results in this study. On a broad scale, severe burn patients with a greater than 20% TBSA who received NSAID treatment during the first week had a decreased risk of developing coagulopathy. When considering the individual days, the day of and day following admission revealed that NSAIDs had their greatest benefit during the first 3 days. Finally, NSAID use showed other benefits, with decreased sepsis, thrombocytopaenia and death.

The pathophysiology behind the development of coagulopathy in severe burns is not well understood, but is thought to be a potential consequence of the haemodilution associated with large volumes of fluid resuscitation and hypothermia [31]. Thus, it is crucial to differentiate hypo-coagulable burn-induced coagulopathy from iatrogenic coagulopathy arising from resuscitation. In this study, we did not match transfusion fluid volumes; however, the volume of fluid resuscitation is usually given according to injury severity [11]. In comparing patients at 10% TBSA intervals, we found the risk of a higher INR decreased in severe burn patients on NSAID treatment.

**Figure 5:**
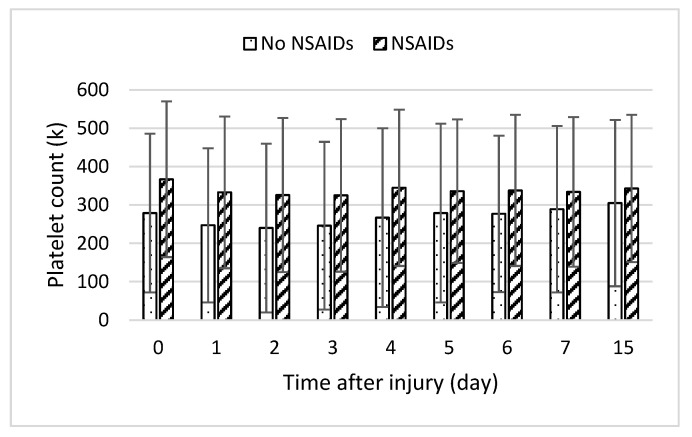
Daily platelet lab counts following burn injury in patients with medication (slant bar) and without medication (dot bar) treatment. Values presented as mean ± SD.

In addition, endothelial damage and the stimulation of the inflammatory response could precipitate coagulation abnormalities. Inflammatory responses lead to hypo-coagulation through the inhibition of fibrinolysis and stimulation of the plasminogen activator inhibitor-1 (PAI-1) by IL-6 and TNF-α [32]. Elevated levels of PAI-1 result in increased difficulty in removing thrombi. Furthermore, past studies showed the heparin–antithrombin pathway is downregulated in certain inflammatory states as neutrophil activation and inflammatory cytokines release products that reduce vascular heparin-like molecules [15]. Inflammatory mediators such as IL-6 also stimulate the production of new platelets, which are more thrombogenic than older platelets [33]. NSAIDs inhibit platelet cyclooxygenase with the development of thrombocytopenia [22]. When we examined platelet counts across time, averages were slightly higher in the NSAID group, but this was not statistically significant (Figure 5). Fibrinogen levels were moderately higher in the group taking NSAIDs, in line with the decrease in coagulopathy risk. We were unable to find PAI-1 levels in the database.

Although not significant, the mean C-reactive protein of patients taking NSAIDs was 69.3 mg/L in this study compared to 87.2 mg/L in patients not taking NSAIDs, and cortisol levels in patients not taking NSAIDs were 21.7 mcg/dL compared to 18.2 mcg/dL for patients taking NSAIDs, suggesting the anti-inflammatory tendencies when taking NSAIDs in severe burn patients.

In 2014, Neal et al. identified that the prehospital use of NSAIDs was associated with a decrease in the incidence of trauma-induced coagulopathy, using criteria defined by a laboratory (INR > 1.5) and clinical (transfusion >2 units of fresh frozen plasma or >1 pack of platelets in 6 h) parameters [34]. The study utilized a stepwise logistic regression and found that prehospital NSAID use was associated with a 72% decrease in the risk of developing trauma-induced coagulopathy. This finding was consistent with those found in our study, which also found a decrease in the coagulopathy risk in patients taking NSAIDs within one week after injury, especially during the first 24 h.

Severe burn patients often undergo surgery for wound excision and grafting. The use of NSAIDs in the perioperative period has been scrutinized for potential adverse effects on soft tissue healing. A systematic review by Constantinescu et al. (2019) assessed the impact of NSAIDs on clinical outcomes after orthopaedic procedures, revealing no significant adverse effects on healing. This suggests that the use of NSAIDs does not compromise recovery [35]. On the other hand, Al-Waeli et al. (2020) investigated the timing of NSAID administration and its effects on postoperative pain and healing. Their findings suggest that the timing of NSAID administration according to the circadian rhythm enhanced postoperative recovery, including in the context of burn injuries [36]. This research pointed to the possibility of optimizing NSAID therapy in terms of the timing of administration to improve outcomes in burn patients.

However, the use of NSAIDs is not without risks. Studies, such as those by Cooper et al. (2019) and Bindu et al. (2020), showed adverse effects, including gastrointestinal, cardiovascular, renal and hepatic complications, particularly in the elderly and those with comorbid conditions [26,37]. Specifically in our study, we examined stroke risk with NSAIDs due to its known cardiovascular complications in previous studies. Ghosh et al. (2015) discussed the role of NSAID-induced reactive oxygen species in cardiovascular diseases, highlighting the adverse effects of NSAIDs, including the increased risk of heart attack and stroke [38]. Fortunately, in this study, we found no increased risk of stroke in the week following burn injury. Ultimately, these findings underscored the necessity of a cautious and judicious approach to NSAID administration in burn patients, particularly those with pre-existing comorbidities or those who are at increased risk of organ damage.

The protection offered by NSAIDs was significant when given the same day and first day following burn injury, which is during the acute resuscitation phase with a hyperinflammatory response when the risk of developing coagulopathy is at its greatest [39]. Severe burn patients are often hyperfibrinolytic the day following burn injury, with increased levels of the tissue-type plasminogen activator. Furthermore, patients are at an increased risk for developing shock because of the depletion of intravascular volume, elevated systemic vascular resistance and depressed cardiac output. Decreased cardiac output along with hepatic and renal flow may decrease drug elimination from the liver and kidneys, which would allow NSAIDs to stay in circulation for an extended period of time [40].

Other secondary protective outcomes of NSAIDs were also analysed, including sepsis, thrombocytopenia and mortality. A recent study from Kim et al. examined the relationship between prior NSAID use and mortality in patients undergoing surgery for abdominal sepsis [41]. They found that those with a prior history of NSAID use had a higher 60-day survival rate than those who did not. This was consistent with the results from our findings, in which mortality was reduced as well in burn patients with NSAID use. From these studies, it appears that this drug class confers a mortality benefit on critically ill patients, although the exact mechanism of this benefit has yet to be elucidated.

In this study, we did not differentiate the individual role of single NSAIDs. Furthermore, in this study, we found aspirin and ibuprofen to be the top two most used NSAIDs in severe burn patients. Patients may receive a combination of NSAIDs during treatment to enhance the anti-inflammatory and pain reduction effects. In fact, we found overlapping use of NSAIDs in this study (Table 1). NSAIDs are usually taken orally, with the specific dosage depending on the drug [20]. The effects usually last multiple hours. Some NSAIDs can also be administered intravenously; IV ibuprofen can be given as a 30 min infusion. The dosage and route of administration could be further studied to evaluate their efficacy in burn patients. A future study could also be conducted to differentiate the effects of individual drugs through a prospective study. Different NSAIDs have distinct half-lives as well, which may cause changes in dosing frequency and even potentially efficacy.

This study, while offering valuable insights into the protective effects of NSAIDs on coagulopathy in severe burn patients, acknowledges several limitations that warrant consideration. Firstly, the potential inclusion of polytrauma patients, who may have sustained additional injuries such as haemorrhage or blunt/penetrating injuries, could have contributed to the observed coagulopathy, independent of the burn injury itself. Such injuries could introduce variability in coagulopathy outcomes, underscoring the complexity of managing burn patients with concomitant traumas. Secondly, the prehospital administration of NSAIDs, either as part of emergency care in rural/remote areas or due to long-term use for other conditions, was not controlled for in this study. This factor could have influenced the baseline coagulation status of patients upon hospital admission. Third, the reliance on the INR as a primary metric for diagnosing burn-induced coagulopathy may not have fully captured the nuanced alterations in coagulation status post-burn injury. Alternative or supplementary diagnostic markers may have provided a more comprehensive understanding of coagulopathy in this patient population. Finally, the study acknowledges the variation in volume resuscitation practices, which were influenced by factors such as patient age (paediatric vs. adult) and injury severity. This variation introduced an additional layer of complexity in distinguishing between injury-induced coagulopathy and resuscitation-induced coagulopathy.

The reduction in coagulopathy risk suggested a pivotal role for NSAIDs in not only improving immediate patient outcomes, but also in potentially reducing the long-term sequelae associated with severe burns. This insight invites a multidisciplinary approach to burn treatment, integrating pharmacological strategies with surgical and rehabilitative care to optimize healing and recovery. Future studies should aim to delineate the mechanisms by which NSAIDs confer their protective effects in burn injuries, exploring their interaction with other treatments and their impact on the overall rehabilitation process. This approach would ensure that the benefits of NSAIDs are utilized to their fullest, contributing to a more refined and effective treatment strategy for burn victims, ultimately leading to improved survival rates and quality of life post-injury.

## 5. Conclusions

In conclusion, we demonstrated that NSAID use was associated with a decreased risk of developing coagulopathy in the severely burned along with additional clinical benefits. We aimed to analyse the impact of NSAIDs in the development of coagulopathy in severe burn patients across various parameters, including the timeline of administration and %TBSA. As shown in this study, NSAIDs decreased the risk of coagulopathy, protective even at higher ranges of %TBSA. In addition, NSAIDs were also shown to provide benefit over comparable analgesics, specifically acetaminophen.

These haematological benefits of NSAIDs were further supported by the decreased risk of thrombocytopaenia, disseminated intravascular coagulation and purpura, along with the decreased risk of sepsis and mortality. We found no increased risk of the development of stroke or haematuria in these patients given NSAIDs, which are commonly feared adverse effects. Therefore, with all of the protective effects in mind, we propose that hospital systems should consider administering NSAIDs to patients undergoing burn resuscitation, particularly during the critical initial days following injury.

## Figures and Tables

**Table 1:** Burn patients with NSAIDs.

NSAIDS	Code	Patient (n)	Patient (%)
Ibuprofen	CN104-5640	648	79%
Oxaprozin	MS102-32613	10	1%
Indomethacin	MS102-5781	12	1%
Aspirin	BL117-1191	322	39%
Diclofenac	MS102-3355	47	6%
Celecoxib	MS102-140587	47	6%
Naproxen	MS102-7258	63	8%

**Table 2:** Demographics of severe burn patients with and without NSAID treatment. * *p* < 0.05; significance measured through chi-squared tests, ** *p* < 0.0001.

Demographics of Each Cohort		NSAIDs	No NSAIDs
Patients	(n)	837	1036
Age	(Mean ± SD)	41 ± 21.6	46.6 ± 21.9 **
Sex	Male	622	747
	Female	214	278
Ethnicity	Not Hispanic	592	677 *
	Unknown	174	285 **
	Hispanic	70	63 *
Race	White	532	621
	African American	127	179

**Table 3:** BIC risk of NSAIDs vs. no NSAIDs and acetaminophen through measures of association to determine significance.

BIC Rate	Odds Ratio	95% CI	Z-Score	*p*-Value
NSAIDs vs. no NSAIDs	0.453	(0.349, 0.586)	−6.068	<0.0001
NSAIDs vs. acetaminophen	0.445	(0.339, 0.586)	−5.836	<0.0001

**Table 4:** The comorbidities and mortality risks between patients prescribed NSAIDs vs. no NSAIDs. DIC—disseminated intravascular coagulation.

BIC Rate	Odds Ratio	95% CI	Z-Score	*p*-Value
DIC	0.41	(0.195, 0.862)	−2.425	0.0153
Purpura	0.583	(0.399, 0.852)	−2.818	0.0048
Sepsis	0.556	(0.368, 0.838)	−2.837	0.0046
Thrombocytopenia	0.435	(0.274, 0.69)	−3.624	0.0003
Mortality	0.196	(0.14, 0.275)	−10.146	<0.0001

## Data Availability

All relevant data are available within the presented manuscript. Any material and information generated during the study can be available for sharing with other researchers under appropriate institutional agreements. Any inquiries should be directed to the corresponding author Dr. Song.
